# Proteomic Validation of Multifunctional Molecules in Mesenchymal Stem Cells Derived from Human Bone Marrow, Umbilical Cord Blood and Peripheral Blood

**DOI:** 10.1371/journal.pone.0032350

**Published:** 2012-05-16

**Authors:** Jumi Kim, Jeong Min Shin, Young Joo Jeon, Hyung Min Chung, Jung-Il Chae

**Affiliations:** 1 CHA Bio & Diostech Co., Ltd., Seoul, Korea; 2 Graduate School of Life Science, CHA Stem Cell Institute, College of Medicine, CHA University, Seoul, Korea; 3 Department of Oral Pharmacology, School of Dentistry, Brain Korea 21 Project, Chonbuk National University, Jeonju, Korea; RWTH Aachen University Medical School, Germany

## Abstract

Mesenchymal stem cells (MSCs) are one of the most attractive therapeutic resources in clinical application owing to their multipotent capability, which means that cells can differentiate into various mesenchymal tissues such as bone, cartilage, fat, tendon, muscle and marrow stroma. Depending on the cellular source, MSCs exhibit different application potentials according to their different in vivo functions, despite similar phenotypic and cytological characteristics. To understand the different molecular conditions that govern the different application or differentiation potential of each MSC according to cellular source, we generated a proteome reference map of MSCs obtained from bone marrow (BM), umbilical cord blood (CB) and peripheral blood (PB). We identified approximately 30 differentially regulated (or expressed) proteins. Most up-regulated proteins show a cytoskeletal and antioxidant or detoxification role according to their functional involvement. Additionally, these proteins are involved in the increase of cell viability, engraftment and migration in pathological conditions in vivo. In summary, we examined differentially expressed key regulatory factors of MSCs obtained from several cellular sources, demonstrated their differentially expressed proteome profiles and discussed their functional role in specific pathological conditions. With respect to the field of cell therapy, it may be particularly crucial to determine the most suitable cell sources according to target disease.

## Introduction

Recently, pluripotent stem cells obtained from fetal tissue or embryos have been a focus of research because of their ability to give rise to a variety of differentiated cell types [1]. Accordingly, many adult stem cell populations are also widely investigated for clinical application in the regenerative medicine field [2], [3]. Among them, mesenchymal stem cells (MSCs) have been recognized as a representative stem cell population present in adult tissue [4].

In 1976, Friedenstein et al. were the first to isolate MSCs from bone marrow (BM-MSCs), a well-known stem cell reservoir, taking advantage of their property of adhering to plastic dishes [5]. The authors demonstrated that the MSCs grew as foci with a fibroblast-like morphology, or colony-forming unit-fibroblasts (CFU-F). In addition, the surface-marker expression profile was verified to be positive for mesenchymal antigens (e.g., CD105, CD13, CD31, and STRO-1) and matrix receptors (e.g., CD44, CD29, and CD73) and negative for hematopoietic markers (e.g., CD34, CD45, and CD14) [6], [7], [8].

In addition to these phenotypic characteristics, MSCs also retain the potential for self-renewal, a high proliferation rate in the presence of defined growth factors and multipotent capacity, which contributes to the regeneration of mesenchymal tissues such as bone, cartilage, muscle, ligament, tendon, adipose and stroma [9], [10].

Owing to their multipotent capacity, BM-MSCs have been investigated since their discovery as promising candidates for use in new cell-based regenerative therapies [11]. However, it is necessary to consider alternative cellular sources for isolating MSCs because of the highly invasive method needed to obtain bone marrow. Therefore, MSCs from different sources have been actively studied; these sources include fatty tissue, placenta, umbilical cord blood, peripheral blood, the pancreas, dental pulp and synovial fluid [12], [13], [14], [15].

MSCs obtained from different sources have been assumed to exhibit similar phenotypic characteristics, irrespective of their original source, as they all have self-renewal properties with respect to common surface epitopes as well as multi-differentiation potential. However, there is currently little information available regarding the mechanisms that govern their involvement in differentiation or in vivo functions [16], 17.

A detailed understanding of the molecular expression profile governing different MSC applications according to their cellular sources is essential for discovering the optimal cell type for clinical use.

Gene expression analyses, such as microarray or DNA chip array, should help in the discovery and elucidation of signaling pathways and molecular mechanisms. However, the gene expression profile does not fully match the functional protein expression profile [18].

In contrast to the transcriptome, proteome analysis can elucidate important components of the proteome, such as protein amount, stability, subcellular localization in a specific cell type or organelle, post-translational modifications during specific developmental and physiological stages and interactions at the protein level [19], [20], [21].

At present, two-dimensional gel electrophoresis (2-DE) and non-2-DE-based approaches are broadly applied to proteomic analyses. Proteome mapping serves as a starting point for building a comprehensive database of the stem cell proteome. Proteomics based on mass spectrometry (MS) has proven extremely useful for analyzing complex protein expression patterns and, when applied quantitatively, can be used to resolve subtle differences across samples.

Several research groups have used proteomics to identify stem cell-specific proteins in mouse ESCs (mESCs), human ESCs (hESCs), human umbilical cord blood (UCB) MSCs, BM-MSCs, rat NSCs and human NSCs [20], [21], [22], [23].

Applying proteomics to investigate the programs that control cell fate should provide valuable insight in understanding how the factors determining their potentially differing applications and which cell type is the most optimal cellular source in specific pathological conditions.

In this study, we isolated MSCs from umbilical cord blood (CB-MSC) and peripheral blood (PB-MSC), which are morphologically and immune-phenotypically similar to MSCs obtained from the BM (BM-MSC). We then compared the differentially expressed protein profiles of BM-MSC, CB-MSC and PB-MSC to verify key regulatory factors that govern potentially different applications using 2-DE-based proteomic analysis tools. According to their specific protein expression profile, we suggested a candidate for the most optimal cellular source in specific pathological conditions in the context of the regenerative medicine field.

On the basis of proteome analysis results, we suggested the strong possibility that BM-MSCs and CB-MSCs have higher viability with respect to ischemic disease than PB-MSCs owing to their antioxidant and detoxification roles.

Also, we examined abundantly differentially expressed key regulatory proteins such as carbonyl reductase 1 (CBR1), ornithine aminotransferase (OAT), HSPB1 (HSP27), PDZ domain-containing protein GIPC1 (GIPC1) and PSAT1. Interestingly, the expression of these proteins exhibited specific expression patterns when they differentiated into adipocyte and osteoblast, regardless of the expression pattern in the undifferentiated stage.

The discovery of the molecular expression associated with specific functions and lineage differentiation capacities should contribute valuably to the field of cell therapy by providing information about the most suitable cell source to the target disease. Therefore, our results provide useful information on the selection of the optimal mesenchymal tissue as a primary source of MSCs for certain purposes.

## Results

### Phenotypic characterization of MSCs obtained from cord blood, peripheral blood and bone marrow

**Figure 1:**
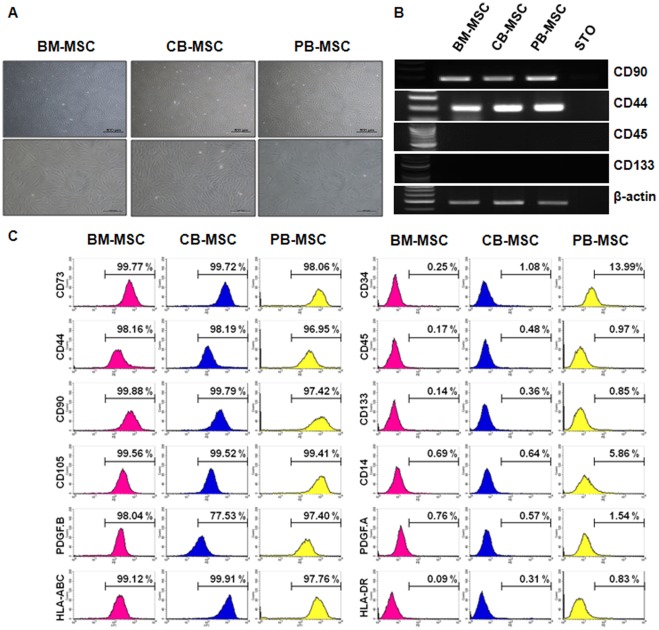
Characterization of MSCs derived from BM, CB and PB. (A) Morphology of cultured BM-MSCs, CB-MSCs and PB-MSCs. MSCs showed fibroblastic-like morphology. Magnification: ×40, ×100. (B) RT-PCR analyses for genes expressed in MSCs. Oct4 and Nanog are undifferentiated stem cell markers. CD90 is a positive marker of MSCs. CD133 is a negative marker of MSCs. (C) Flow cytometric analysis of cell surface markers of MSC from BM, CB and PB. MSCs expressed CD90, and CD44 but not CD34, CD45, and CD133. CD34, CD45, and CD133 are negative markers, and CD90, and CD44 are positive markers.

To confirm the phenotypic characteristics of cells obtained from CB, PB and BM as MSCs, we performed morphological, RT-PCR and FACs analysis. Three types of MSCs obtained from three different cellular sources retained similar phenotypic characteristics (Fig. 1).

Immediately after the isolation of CB-MSCs and PB-MSCs using the Ficoll reagent, adherent colony-forming cells appeared (see passage 0, Figure S1). After 14 days, proliferating MSCs exhibited fibroblastic and spindle-like morphology after passage 3, much like the purchased BM-MSCs (Fig. 1A).

Then, we compared the general marker expression profiles of CB-MSCs, PB-MSCs and BM-MSCs at the RNA and protein levels. In RT-PCR analysis, mesenchymal markers CD90 and CD44 were expressed very strongly, whereas hemangioblast marker CD133 and hematopoietic cell marker CD45 were not detected (Fig. 1B).

In surface-marker expression analysis using the FACs analyzer, CD34, CD45 (both hematopoietic cell markers) and CD133 were expressed negatively in all paired samples, whereas they were extremely positive for CD73 (adhesion marker, over 98%), CD44 (adhesion marker, over 97%), CD90 (mesenchymal marker, over 95%), CD105 (mesenchymal marker, over 99%), PDGF-B (over 77%) and HLA-ABC (over 97%) (Fig. 1C).

And cell growth rate is similar in all three cells (Fig. S2)

As the result, we confirmed that the isolated cells from CB and PB in this study are also MSCs and are similar to purchased BM-MSCs.

### *In vitro* differentiation of MSCs derived from different cellular sources

To investigate the differentiation capacity of MSCs derived from different sources *in vitro*, MSCs were cultured in differentiating induction medium for 4 weeks to induce adipogenic, osteogenic and chondrogenic differentiation. Then, the differentiation capacity of MSCs was characterized by immunocytochemistry and real-time RT-PCR.

**Figure 2:**
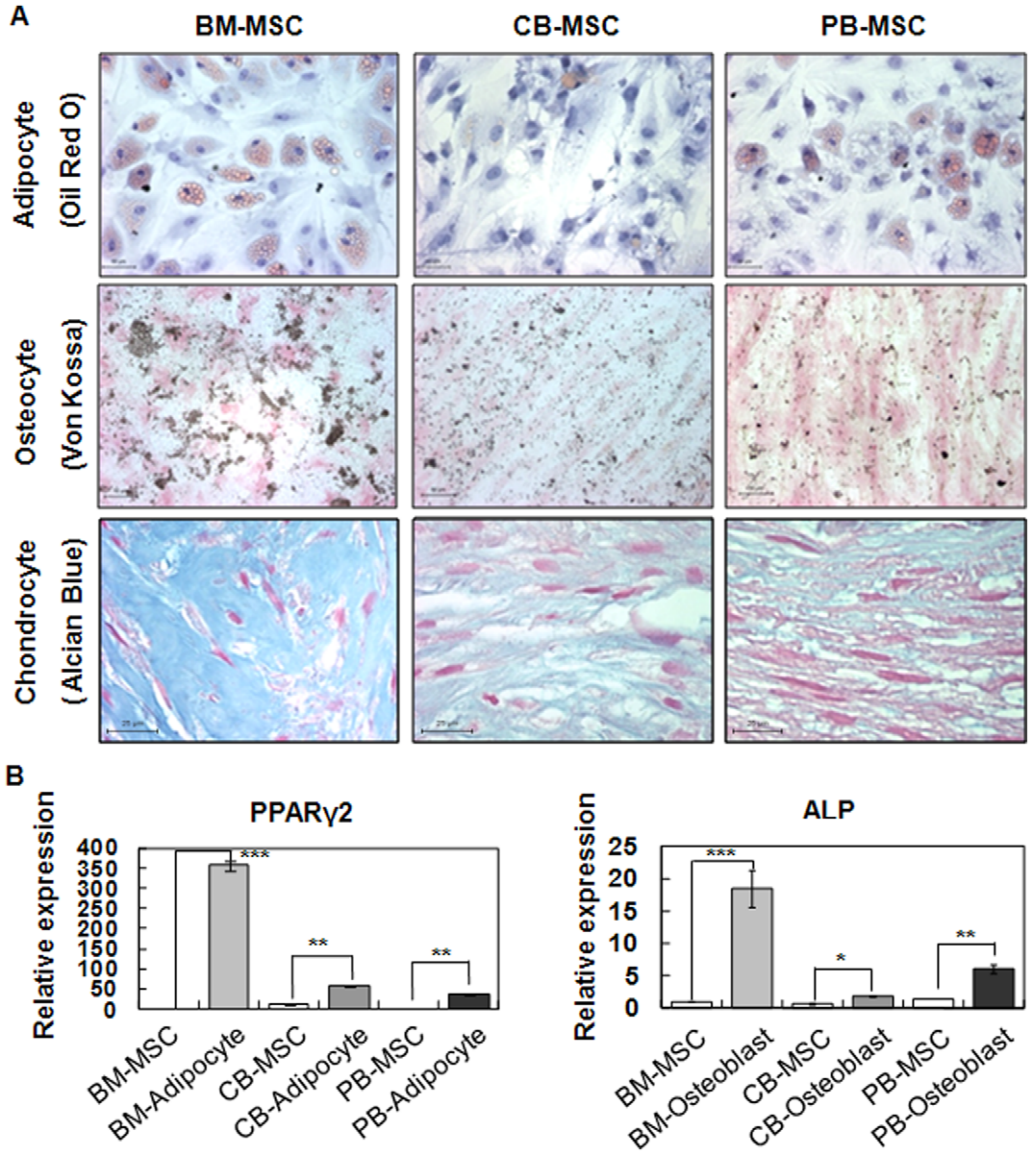
Differentiation potential of MSCs isolated from BM, CB and PB (in vitro differentiation of MSC cultures). (A) Adipogenesis of BM-MSCs, CB-MSCs, and PB-MSCs. Cells were stained with Oil Red O after 3 weeksin induction culture. The lipid globules of adipocytes were stained red. Scale bar, 50 µm. Osteogenesis of BM-MSCs, CB-MSCs, and PB-MSCs. Cells were stained with Von Kossa after osteogenic induction for 3 weeks. Calcium deposits of osteocytes were stained in brown. Scale bar, 50 µm. Chondrogenesis of BM-MSCs, CB-MSCs, and PB-MSCs. Cells were stained with Alcian Blue after chondrogenic induction for 3 weeks. Proteoglycans deposits of chondrocytes were stained blue. Scale bar, 25 µm. (B) Relative expression of adipogenic and osteogenic markers in BM-MSCs, CB-MSCs, and PB-MSCs before (d0, white bars) or after (d22, gray bars) induction of differentiation. Data (n = 3) are presented as the mean S.D., and asterisks indicate statistically significant values. The significance of differences was evaluated by independent samples Student's *t*-test (SAS version 8.0, Cary, NC, USA). *p<0.05, **p<0.01 and ***p<0.001.

The adipogenic differentiation potential of MSCs was determined by the lipid globule adipocyte colony formation and Oil Red O positive staining when MSCs were cultured in adipogenic differentiation media. The lipid globule adipocyte colonies appeared after day 5, day 14 and day 22 in BM-MSCs, PB-MSCs and CB-MSCs, respectively (data not shown). After 4 weeks, the adipocyte colonies were approximately the same size in the BM-MSC- and PB-MSC-derived adipocytes, but CB-MSCs produced fewer and smaller lipid vacuoles in contrast to those of BM-MSCs and PB-MSCs. Additionally, the rate of Oil Red O-positive colonies among cultured cell populations in differentiating induction medium was varied. The rate of Oil Red O-positive colonies was highest in BM-MSC and lower in PB-MSC. CB-MSCs showed the lowest recorded rate of Oil Red O-positive colony formation (Fig. 2A, upper panel).

When MSCs were cultured in an osteogenic medium, MSCs displayed calcification colonies and a positive population under Von Kossa staining of the mineralized matrix. To distinguish the osteogenic differentiated cell population from each MSCs, we performed Von Kassa immunostaining. Among the three different MSCs, the rate of Von Kassa positive colonies was higher for BM-MSCs than CB-MSCs and PB-MSCs (Fig. 2A, middle panel).

The chondrogenic differentiation of MSCs was induced via the pellet culture method and confirmed by proteoglycan deposition using Acian Blue staining when MSCs were cultured in chondrogenic differentiation media. Chondrogenic differentiated pellets from BM-MSCs were clearly positive under Acian Blue staining, whereas staining regions were very dim for CB-MSCs and PB-MSCs.

To quantify the differentiation capacities of the three cell types, real-time RT-PCR was performed to measure the expression of genes specifically involved in adipocyte and osteoblast differentiation. The relative expression levels of mRNA for PPARγ2 and ALP were used as markers of adipogenic and osteogenic differentiation, respectively. The RNA expression of PPARγ2 and ALP were significantly increased in adipogenic and osteogenic differentiated cells from BM-MSCs, indicating that these cells underwent adipogenic or osteogenic differentiation better than other types of derived MSCs (Fig. 2. B).

These data indicated that BM-MSCs have a higher potential to differentiate into adipocytes, osteocytes and chondrogenesis *in vitro* than do other MSCs. As a result, despite the similar characteristics of MSCs derived from different sources in the undifferentiating state, MSCs retain variable differentiation potentials after inducing differentiation.

### The comparative proteomic profile of CB-MSCs, PB-MSCs and BM-MSCs

To study the mechanism for elucidating the fundamental differences among MSCs obtained from different cellular sources, we harvested CB-MSCs, PB-MSCs and BM-MSCs at passage 2 and performed proteomic analysis using 2-DE analysis coupled with mass spectrometry protein identification. Multiple gels from the three independent replications were performed to certify the reproducibility of the protein homogenates on 2-DE gels. The analytical gels were visualized using silver staining.

**Figure 3:**
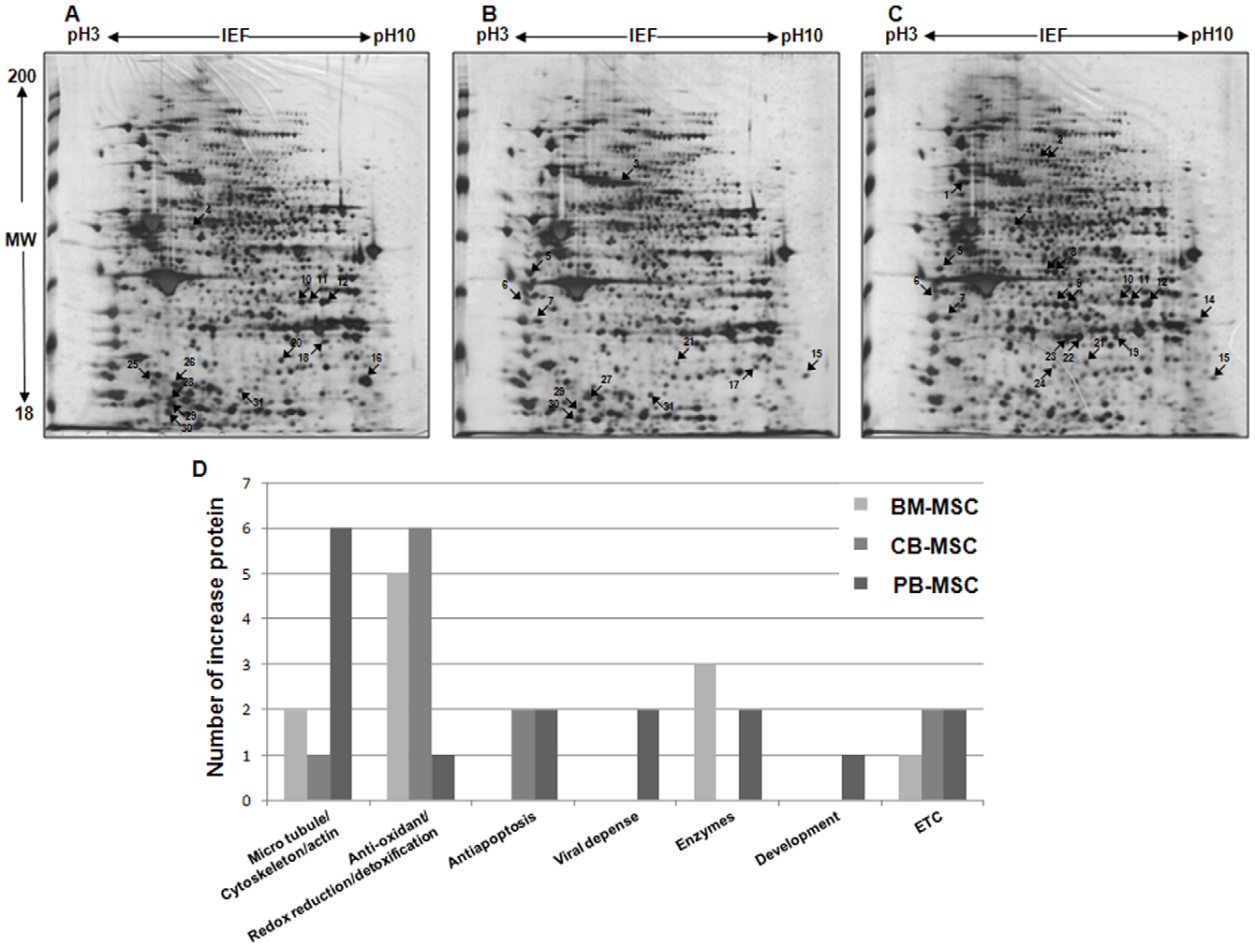
2-DE gel electrophoresis and Classification of differentially regulated proteins from proteomic analysis. Proteins were isolated from total protein of MSCs loaded onto the 2-DE gel electrophoresis. First dimension was 1 cm with a pH of 3 to 10 with NL IPG, and the second dimension was 10% gels (A: BM-MSCs, B: CB-MSCs, C: PB-MSCs). They were visualized using silver staining. The number indicates the position of up-regulated protein spots in the 2-DE pattern. (D) Ontological classification of differentially regulated proteins in terms of molecular functions using the Gene Ontology (http://www.geneontology.org) and UniProt (http://www.expasy.uniprot.org) websites. The composition of the identified proteins is presented as numbers for all individual identified proteins.

As shown in Figure 3, (pH 3–10, molecular weight 18–200 kDa) and despite similar general protein expression patterns in the three types of MSCs, 11 of the proteins were abundantly up-regulated in BM-MSCs and CB-MSCs, and 16 of the proteins were up-regulated in PB-MSCs (Fig. 3. A, B and C). Consequently, differentially expressed proteins were identified using LC-MS/MS. Identified proteins were listed in Table S3, S4 and S5.

### Classification of differentially regulated proteins based on proteomic analysis

The list of identified proteins was then used to categorize their functional involvement as documented in Gene Ontology (http://www.geneontology.org) and UniProt (http://www.expasy.uniprot.org) websites; the results were displayed as a graph (Fig. 3. D). A database search and functional exploration of these differentially expressed proteins showed that these proteins have different roles that can be broken down into five classification based on their functions. This includes cytoskeleton proteins (i.e., tubulin alpha 1 C chain, annexin A2, Lamin B1, PDZ domain-containing protein GIPC 1 and vimentin actin), antioxidant and detoxification proteins (i.e., carbonyl reductase [NADPH], heat shock proteins beta 1 (HSP 27), glutathione S-transferase Mu3, glutathione S-transferase omega-1, S-formylglutathione hydrolase, anexxin A1, and chloride intracellular channel protein 4), anti-apoptotic proteins (i.e., isoform 1 of nucleophosmin and 40S ribosomal protein S3), viral defense-related proteins (i.e., interferon-induced GTP binding protein Mx 1 and ornithine aminotransferase), viral defense-related enzymes (i.e., isoform 1 of phosphoserine aminotransferase, L-lactate dehydrogenase, inositol monophosphatase and nicotinamide N-methyltransferase) and development-related protein (i.e., isoform 1 of axin interactor and dorsalization-associated protein).

The largest category was the antioxidant and detoxification group (26.92 %, or 2/26), whereas the second largest group was the cytoskeletal category (23.08 %, or 2/26).

However, the distribution of the categories was very different based on their cell source. Among BM-MSCs and CB-MSCs, antioxidant and detoxification proteins were the largest category. However, in the case of cytoskeletal proteins, PB-MSCs were the most represented MSCs, although differences in the absolute number of proteins were present.

### Validation of functional relevance of proteome expression profiled with therapeutic capacity of BM-, CB- and PB-MSC

To investigate the functional relevance between protein expression profile, which involved in antioxidant, detoxification and cytoskeleton function, and each cell lines including BM-, CB- and PB-MSC, we generated wound disease model and examined the therapeutic capacity of each cell lines for 14 days.

Wound model is suitable disease model for comparison of functional behavior of each cells in ischemic tissue, because wound regions is rapidly fall into deep ischemic condition and wound is one of the most frequently developed disease associated with several kinds of ischemic disease model.

After generation of wound disease model as described in material and method section, we applied 3×10^6^ cells of each cells, including BM-, CB- and PB-MSCs, to excisional wounds via subcutaneous injection around the wound site. Vehicle control medium were used as the negative controls. Wound healing was then evaluated by photometric analysis of the wound tissue using the calculating of wound closing rate as a relative value using an image analysis tool (Image J) over 14 days.

**Figure 4:**
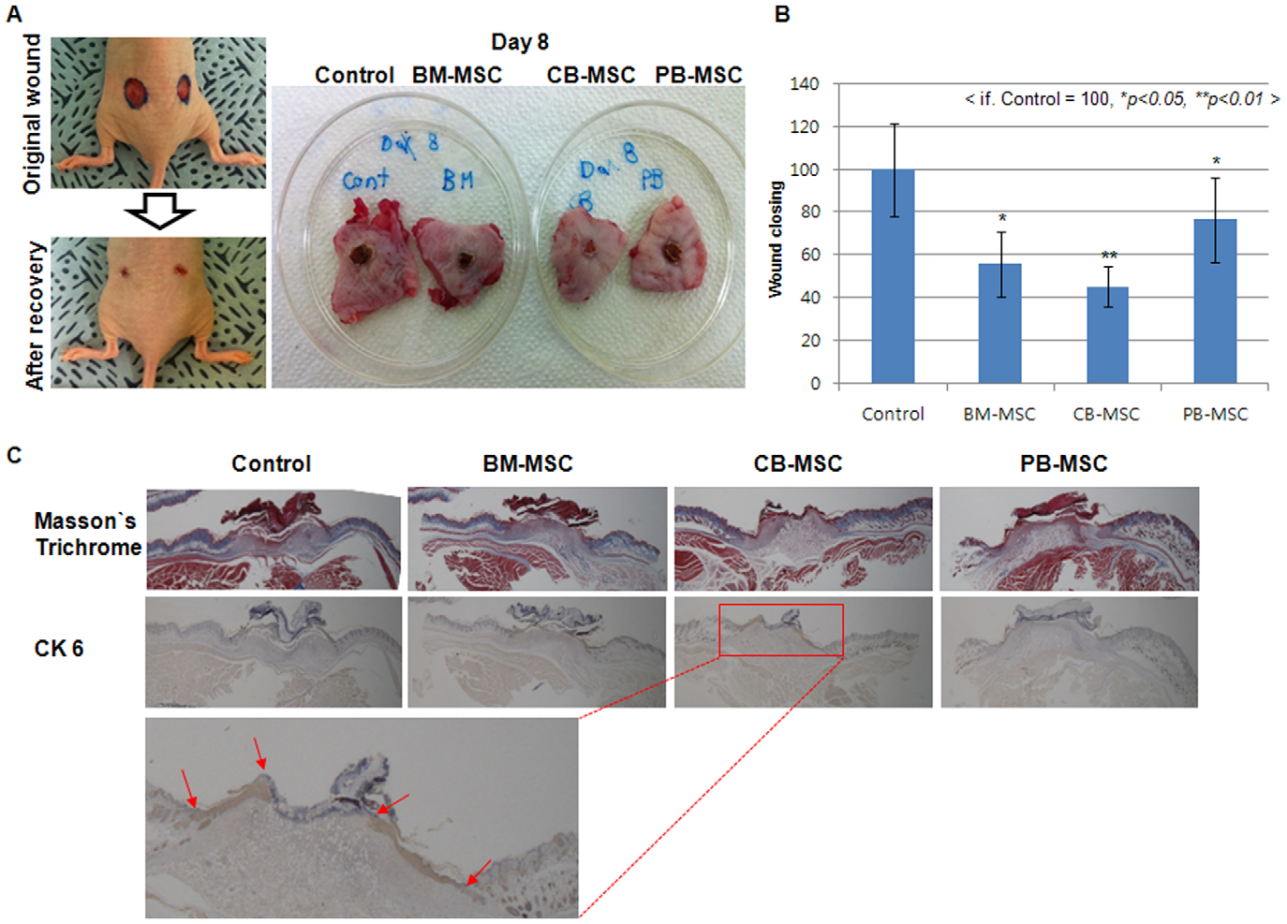
Comparison of the therapeutic effect of BM-, CB- and PB-MSCs on wound disease model mouse. (A) Representative photographs of wounds after treatment with control vehicle medium (medium control), BM-MSC (3×10^6^ cells), CB-MSC (3×10^6^ cells) and PB-MSC (3×10^6^ cells) by subcutaneous injection around the wound site. (B) Measurement of wound closing at different days. Data (reach group: n = 6) are presented as the mean S.D., and asterisks indicate statistically significant values. The significance of differences was evaluated by Student's *t*-test (SAS version 8.0, Cary, NC, USA). *p<0.05, **p<0.01 (C) CK 6 staining of control medium, BM-MSC (3×10^6^ cells), CB-MSC (3×10^6^ cells) and PB-MSC (3×10^6^ cells) treatment groups on days 8. Red arrows mean CK-6 positive re-epithelilazed region in CB-MSC transplantation group.

Immediately after wounding with a biopsy punch (6 mm) device on the both side of dorsal region, wounds displayed an epithelial gap of 6.67±0.27 mm, and the wound temporally became wider because of wound contraction (data not shown). After three days, wound closing could be detected as photometric analysis, and the difference of wound closing rate between different cell lines, including BM-, CB- and PB-MSCs, was more evidently after 8 days of cell transplantation (Fig. 4).

At 8 days, among three cell line treated group, including BM-, CB- and PB-MSCs, CB-MSC-treated group exhibited a more rapid healing rate than BM- and PB-MSC transplantation group.

In morphological and relatively calculated wound closing rate analysis (P<0.05, n = 6) using photometric result of wound region 8 days after cell transplantation (Fig. 4. A and B), remaining wound size was reached at 58, 42 and 78 in BM-, CB- and PB-MSC transplanted group, respectively (Fig. B, P<0.05, n = 6), when the remaining wound size was relatively calculated as 100 in control vehicle group. These result indicate that the wound closing was proceeded approximately about 42%, 58% and 22% in BM-, CB- and PB-MSC transplanted group, respectively.

We then performed histological analyses on the central sections of the wounds to evaluate the re-formation of granulation tissue on wounded tissue and re-epithelialization at day 8 after cell transplantation.

At day 8 after cell transplantation, the re-formation of granulation tissue in CB-MSC transplanted wounds (red box with dotted line) appeared to be thicker and larger than in BM-MSC, PB-MSC or vehicle medium treated wounds, and it continuously improved day by day (Fig. 4C).

Re-epithelialization, which was examined using CK 6 Ab staining, was similar until day three or four (data not shown), after which re-epithelialization accelerated. At day 8 after treatment, re-epithelialization occurred rapidly in the CB-MSC transplantation group compared with the control groups (Fig. 4D). At day 8 after treatment, scar area was the smallest in the CB-MSC transplanted wound and CK 6-positive epithelial cells covered over half of the wound region. Also, as similar with re-formation of granulation tissue in CB-MSC transplanted wounds, CK 6-positive re-epithelialized tissue in CB-MSC transplanted wounds (red arrow region) appeared to be thicker and larger than in BM-MSC, PB-MSC or vehicle medium treated wounds, and it continuously improved day by day (Fig. 4D).

However, the BM-MSC, PB-MSC transplantation and vehicle control medium-treated groups still showed large scars and incomplete re-epithelialization with the wide wound epithelial gaps.

### Confirmation of anti-oxidant, detoxification and cytoskeleton molecular expression pattern using western blot and real-time RT-PCR analysis

**Figure 5:**
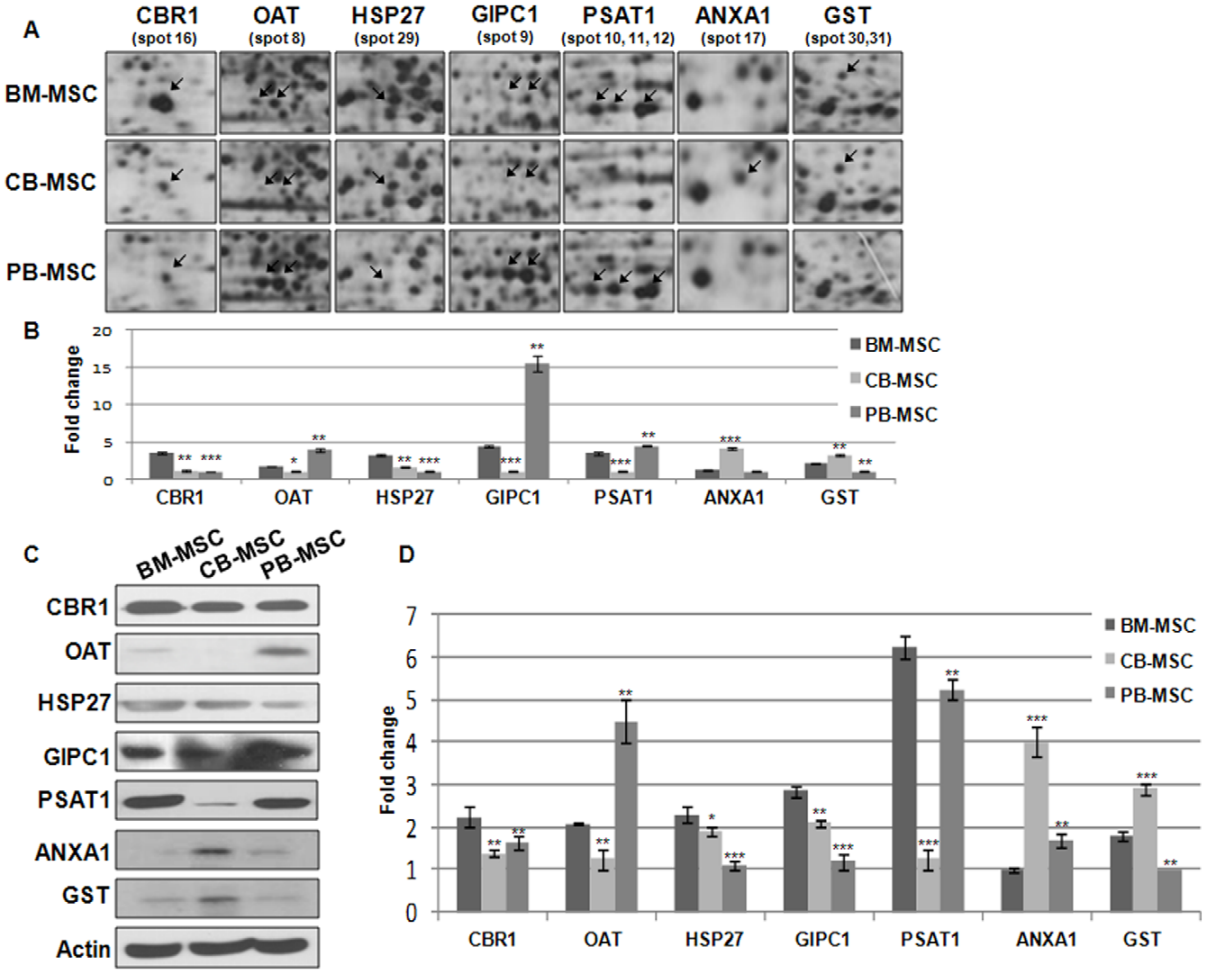
Confirmation of differentially expressed proteins and genes using western blot and real-time RT-PCR analysis. (A) Enlarged images of differentially expressed protein spots among BM-MSCs, CB-MSCs, and PB-MSCs. (B) The amount of protein expression was measured by scanning and the TINA analysis program. (C) Confirmation of differentially expressed proteins in MSCs by Western blot analysis (that is, CBR1, OAT, HSP27, GIPC1, PSAT1, ANXA 1 and GST). Actin was used as an internal control. (D) Real-time RT-PCR analysis of human of CBR1, OAT, HSP27, GIPC1, PSAT1, ANXA 1 and GST in BM-MSCs, CB-MSCs, and PB-MSC. Data (n = 3) are presented as the mean S.D., and asterisks indicate statistically significant values. The significance of differences was evaluated by independent samples Student's *t*-test (SAS version 8.0, Cary, NC, USA). *p<0.05, **p<0.01 and ***p<0.001, compared with BM-MSC.

Proteins were regarded as strikingly differentially expressed across BM-MSCs, CB-MSCs, and PB-MSCs in the undifferentiated stage when the magnitude of difference was greater than 2 fold or more. As a result, five proteins were found to have highly differential expression among MSCs derived from different sources; they included carbonyl reductase 1 (spot 16), OAT (spot 8), HSP27 (spot 29), GIPC1 (spot 9), PSAT1 (spot 10, 11, 12) ANXA 1 (spot 17) and GST (spot 30,31) shown as enlarged protein spots in Fig. 5A. In brief, CBR1 was expressed at a higher level in BM-MSCs as compared with CB-MSCs and PB-MSCs. Both OAT and GIPC1 were expressed at a higher level in PB-MSCs. BM-MSCs and CB-MSC showed a higher expression of HSP27 as compared with PB-MSCs. PSAT1 was expressed in BM-MSCs and PB-MSCs. ANXA 1 and GST were higher in CB-MSC than BM- and PB-MSC (Fig. 5B).

To confirm the results obtained in 2D-DE gels, several proteins, such as CBR1, OAT, HSP27, GIPC1, PSAT1, ANXA 1 and GST from three sources of MSCs were selected for further analyses by using Western blot and real-time RT-PCR analysis.

To quantitatively confirm expression patterns, we performed Western blot analysis and compared the degree of expression using an imaging analysis program (Fig. 5C).

Western blot analysis of undifferentiated BM-MSCs, CB-MSCs and PB-MSCs showed that CBR1 was highly expressed in BM-MSCs as compared with its expression in CB-MSCs and PB-MSCs, whereas OAT and GIPC1 were expressed more in PB-MSCs. PSAT1 was expressed in BM-MSCs and PB-MSCs. HSP27 was highly expressed in BM-MSCs. ANXA 1 and GST were higher in CB-MSC than BM- and PB-MSC, as also shown in the 2-DE results in Fig. 5A and B.

To verify their genomic-level expression, we performed real-time RT-PCR analysis together with western blot analysis. The expression pattern of OAT, HSP 27, GIPC 1 and PSAT 1 across BM-MSCs, CB-MSCs and PB-MSCs were generally comparable to 2-DE and western blot analysis, except for the results for CBR 1. CBR1 was highly expressed in BM-MSCs as compared with its expression in CB-MSCs and PB-MSCs. The expression quantity in CB-MSCs and PB-MSCs was similar in 2-DE and western blot analysis, whereas in CB-MSCs, it was extremely low as compared to PB-MSC according to real-time RT-PCR analysis. ANXA 1 and GST were highly expressed in CB-MSC than BM- and PB-MSC (Fig. 5D).

### Immunofluorescent-staining photomicrography for protein expression patterns and localization

**Figure 6:**
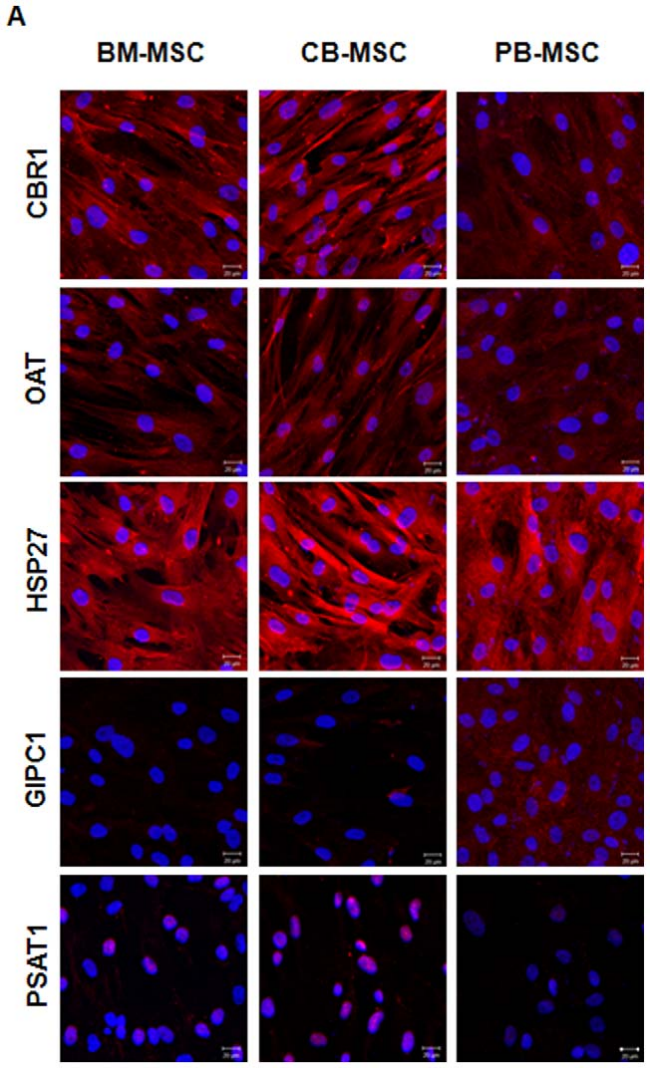
Immunofluorescent-staining photomicrography for protein expressed in undifferentiated BM-MSCs, CB-MSCs, and PB-MSCs. Blue color is DAPI staining. Scale bar: 20 µm.

Immunocytochemical staining also verified the expression patterns and the expression localization of five proteins across the three different MSCs (Fig. 6).

Although quantitative analysis is not enough when using immunocytochemical analysis, the examination of expressing localization is also an important aspect to consider when discussing functional activity.

CBR1 is an oxidoreductase, which plays a role in oxidation reduction. It is localized in cytoplasmic portion of widely distributed tissues in the human body. OAT is an enzyme involved in visual perception; its expression is normally observed in mitochondria in the cytoplasm.

HSP 27 is one of the representative proteins involved in stress resistance and actin organization. HSP 27 is detected in the cytoplasm of several tissues, including skeletal muscle, heart, aorta, large intestine, small intestine, stomach, esophagus and many others. The highest of expression are found in the heart and in tissues composed of striated and smooth muscle.

GIPC 1 is the actin-binding protein that influences endothelial cell migration. GIPC 1 is widely expressed in the cytoplasm of many tissues.

PSAT 1 is expressed in the cytoplasm and nucleus; it is expressed at high levels in the brain, liver, kidney and pancreas, whereas it is very weakly expressed in the thymus, prostate, testis and colon.

According to immunocytochemical analysis in this study, the localization of CBR 1, OAT, HSP 27, GIPC 1 and PSAT 1 across the three types of MSCs was similar to that seen in previous studies. In particular, in CB-MSCs, the localization of PSAT 1 was more concentrated in the nucleus as compared with the BM-MSCs and PB-MSCs. In this study, all protein information was available and searched on NCBI and UniProt KB databases.

### Expression pattern of target genes and proteins during adipogenic and osteogenic differentiation

The relationship between differentiation capacity and the involvement of specific molecules on differentiation is also important to consider. Therefore, we examined molecular expression patterns during differentiation. Additionally, we investigated the involvement of these target proteins, including CBR 1, OAT, HSP 27, GIPC 1 and PSAT 1, during MSC differentiation and verified their protein expression pattern relating to adipogenesis and osteogenesis using real-time RT-PCR analysis and immunocytochemistry.

As compared with undifferentiated MSCs, all proteins were down-regulated during adipogenic and osteogenic differentiation in all three different MSCs (data not shown).

**Figure 7:**
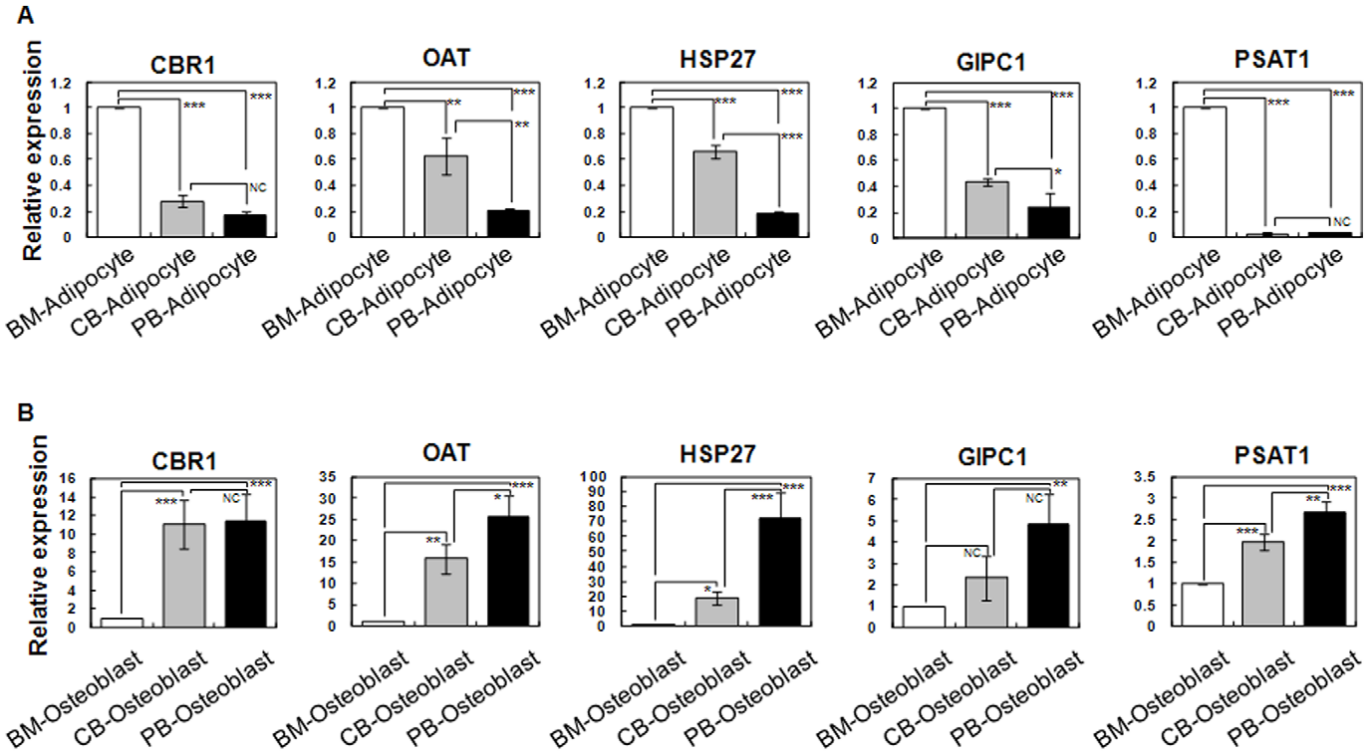
Expression pattern of target genes during adipogenic and osteogenic differentiation using real-time RT-PCR analysis. Data (n = 3) are presented as the mean S.D., and asterisks indicate statistically significant values. The significance of differences was evaluated by paired independent samples Student's *t*-test (SAS version 8.0, Cary, NC, USA). NC = no significant differences, *p<0.05, **p<0.01 and ***p<0.001.

Interestingly, when differentiating into adipocytes and osteocytes, the five target proteins showed specific gene expression patterns, regardless of their cellular source and MSC expression pattern in an undifferentiated state. The expression of all five proteins was highest in BM-MSCs, whereas their expression decreased slightly in CB-MSCs and PB-MSCs. In contrast, the expression of the five proteins slightly increased in CB-MSCs and PB-MSCs as compared BM-MSCs (Fig. 7).

**Figure 8:**
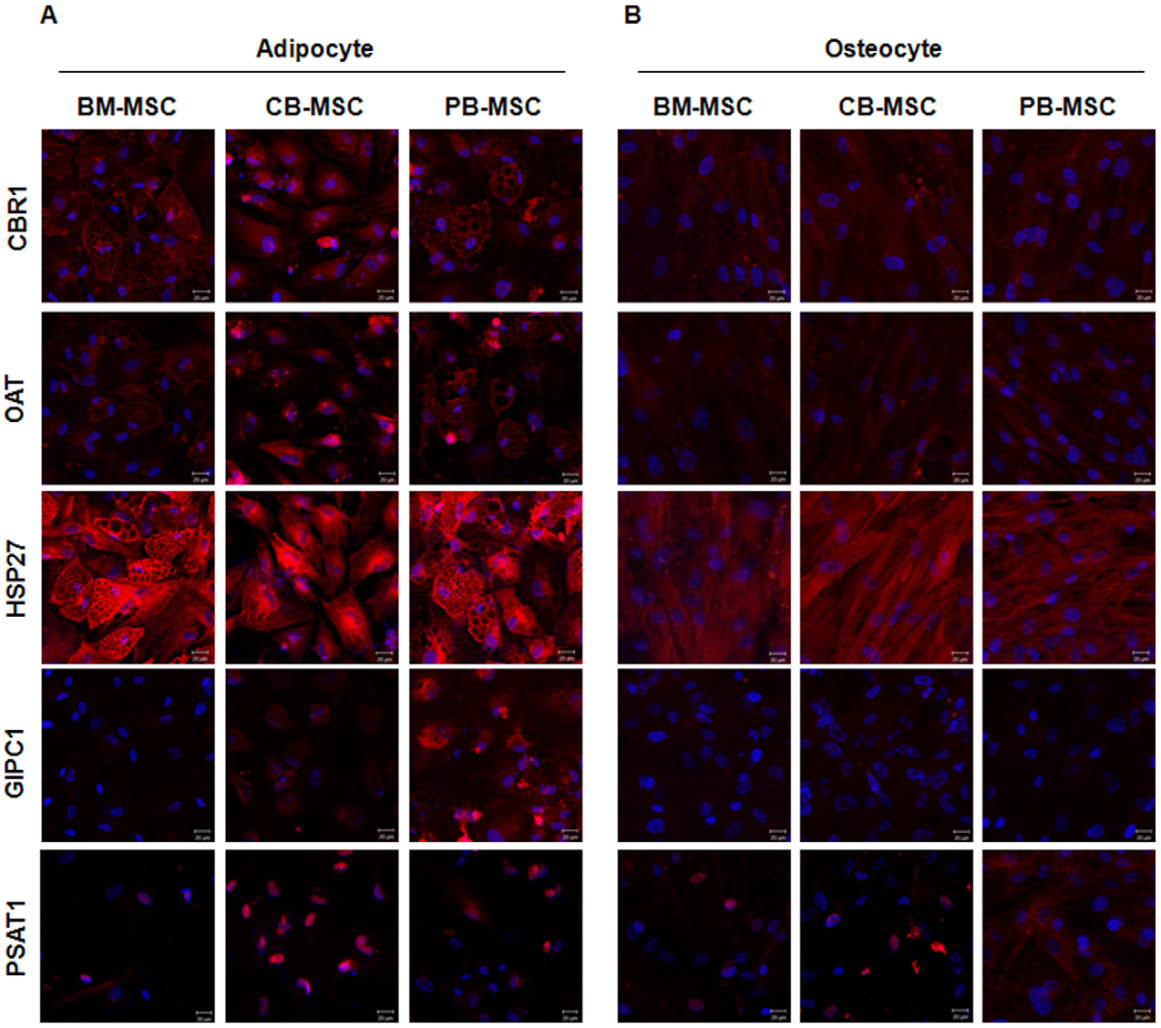
Immunofluorescent-staining photomicrography for protein expressed in differentiated adipocytes and osteoblasts. Blue color is DAPI staining. Scale bar: 20 µm.

To verify their expression localization, we also performed immunostaining analysis after adipogenic and osteogenic differentiation in the three types of MSCs (Fig. 8). Although the expression pattern was dissimilar to the undifferentiating stage of the MSCs, expression localization in the cytoplasm lasted after adipogenic and osteogenic differentiation in the case of CBR1, OAT, HSP 27 and GIPC 1. This was the same result as that for the undifferentiated MSCs. Specifically, in the case of PSAT 1, expression was more concentrated in the nucleus in CB-MSCs after differentiation into adipocytes. Additionally, the localization of PSAT 1 was more robust in the cytoplasm in PB-MSCs after differentiating into osteocytes as compared to the undifferentiated state. Our data revealed cell-specific differences in the protein expression levels between the three cell types under investigation based on their tissue orientation toward adipogenic and osteogenic differentiation.

## Discussion

Since the discovery of MSCs in bone marrow in 1976, MSCs have been shown to have promise for clinical applications due to their accessibility, expandability and multipotentiality [5]. However, the shortage of donor cells and the deeply invasive methods needed to obtain bone marrow has created a major problem in transplantation medicine using BM-MSCs. This problem could be alleviated through the use of other stem cell sources, such as fatty tissue, placenta, umbilical cord blood, peripheral blood, pancreas, dental pulp and synovial fluid. Among them, umbilical cord blood and peripheral blood are considered to be the most suitable stem cell sources because they are easy to obtain through circulation with non-invasive method [6], [9], [13], [14], [15].

During the past decade, there has been an increase in research activity aimed at understanding the cellular and molecular aspects of cell proliferation and differentiation of various sources of stem cells because the molecular events governing their expansion and differentiation remain poorly understood.

Therefore, as a first step to understanding the different molecular expression patterns that govern MSCs function differently based on cellular source, we proteome-profiled MSCs to identify differentially expressed key regulatory factors and discussed their functional role in specific pathological conditions. Such an analysis may be crucial to eventually determine the most suitable cell source for a target disease. This information should thus greatly contribute to the field of cell therapy.

To compare MSCs using proteomic analysis, we derived MSCs from umbilical cord blood and peripheral blood and characterized them in comparison to purchased BM-MSCs. In phenotypic analysis, three different type of MSCs (namely, BM-MSCs, CB-MSCs and PB-MSCs) showed similar fibroblastic and spindle-like morphological appearances. During surface-marker expression analysis using the FACs analyzer, they were shown to retain similar marker expression profiles, which were positive for mesenchymal (CD90) and adhesion markers (CD44) and negative for hematopoietic markers (CD34, CD45 and CD133). BM-MSCs, CB-MSCs and PB-MSCs all exhibited general MSC characteristics in phenotypic analysis in the undifferentiated state, as has been previously reported. However, when MSCs are differentiated into three mature cell types (namely, adipocytes, osteoblasts and chondrocytes), the MSCs showed different differentiation potentials based on cellular source during an analysis to confirm their multipotent ability. Our data indicated that BM-MSCs have a higher potential to undergo adipogenesis, osteogenesis and chondrogenesis than other MSCs. Although MSCs from three different sources were highly similar in the undifferentiated state, these differences in differentiation capacity could be functionally related to the MSC source.

Next, proteomic approaches were applied in order to identify the respective regulatory molecules. The protein expression profiles among BM-MSCs, CB-MSCs and PB-MSCs were compared using 2-DE and combined MS/MS analysis.

Despite the similar general protein expression patterns of the three MSC types, 11 of the proteins were abundantly up-regulated in BM-MSCs and CB-MSCs, and 16 of the proteins were up-regulated in PB-MSCs.

Identified proteins that specifically up-regulated in BM-MSCs, CB-MSCs and PB-MSCs were classified according to their biological processes and molecular functions based on gene ontology. They were also grouped into different categories according to their functions as documented on the Gene Ontology and UniProt websites, including cytoskeleton proteins, antioxidant and detoxification proteins, viral defense-related proteins, enzymes and development-related protein. The protein expression profile is specifically expressed in one cell type or overlapped across the three MSC types. Cytoskeletal proteins were abundantly expressed in PB-MSCs (that is, tubulin alpha 1 C chain, annexin A2, lamin B1, PDZ domain-containing protein GIPC 1 and actin), and some of these proteins were also strongly expressed in BM-MSCs (namely, tubulin alpha 1 C chain and annexin A2). Furthermore, antioxidant and detoxification proteins were abundantly expressed in both BM-MSCs (namely, carbonyl reductase [NADPH], heat shock proteins beta 1 (HSP 27), glutathione S-transferase Mu3 and glutathione S-transferase omega-1) and CB-MSCs (namely, heat shock proteins beta 1 (HSP 27), glutathione S-transferase Mu3, glutathione S-transferase omega-1, S-formylglutathione hydrolase, anexxin A1 and chloride intracellular channel protein 4).

These expression profiles may be correlated with functional behavior in vivo because cytoskeleton and anti-oxidant and detoxification roles are considered to be the most important aspect of cell engraftment and survival in ischemic or damaged tissue regions [24], [25]. Indeed, these are the regions in which cell transplantation of MSCs are performed to cure degenerative diseases, such as cardiovascular or neurodegenerative disease [2], [3]. Cytoskeleton proteins participate in the improvement of cell migration and engraftment, and antioxidant and detoxification proteins enhance cell survival ability under harsh conditions, such as during hypoxia, in the ischemic region [26], [27], [28]. In such conditions, when the protein expression profile corresponds to in vivo functional performance, PB-MSCs will be retain remarkable engraftment and/or migration abilities due to their up-regulated cytoskeleton protein expression. In addition, the high migration ability of PB-MSCs could be a natural result because PB-MSCs are mobilized cell population from bone marrow. Also, CB-MSCs and PB-MSCs could be a more optimal cellular source for vascular or neural degenerative disease caused by hypoxic conditions in the ischemic region of damaged tissue due to their highly up-regulated proteins, which are involved in antioxidant and detoxification function. Moreover, co-transplantation of different MSCs could exhibit both high engraftment and migration ability and antioxidant and detoxification function.

To investigate these points which related to functional relevance between specifically up-regulated molecules and each cell lines including BM-, CB- and PB-MSC, we generated wound disease model and compared their therapeutic capacity in ischemic tissue when cells transplanted. It is possible that comparison of functional behavior of each cells in ischemic tissue on wound healing disease model, because wound is one of the most frequently developed disease associated with several kinds of ischemic disease model.

In comparison, CB-MSC transplanted group was shown the highest therapeutic ability at the point of re-formation of granulation tissue and re-epithelialization on wound site.

Therapeutic effect after stem cell therapy is really closed with cell survival rate and cell engraftment efficacy on the damaged site. However, the statistical analysis of engraftment ratio is very difficult due to the technical limitation and the very extremely low cell engraftment efficacy, because it is well known that cell engraftment efficacy of stem cell therapy is less than 1%. This means that cell survival capacity is one kind of important aspect of healing process.

In this study, anti-oxidant, detoxification and anti-apoptosis related molecules, including heat shock proteins beta 1 (HSP 27), glutathione S-transferase Mu3, glutathione S-transferase omega-1, S-formylglutathione hydrolase, anexxin A1 and chloride intracellular channel protein 4, were specifically up-regulated and the therapeutic effect was the highest in CB-MSC than BM-MSC or PB-MSC.

These result suggest that CB-MSCs could be a more optimal cellular source for ischemic disease caused by hypoxic conditions due to their highly up-regulated proteins, which are involved in antioxidant and detoxification function, and these function have more dominant position in therapeutic capacity than cytoskeleton function, when the protein expression profile corresponds to in vivo functional performance.

Another analytical approach of this study required the use of proteomics to identify the product of cell fate regulation and differentiation of MSCs based on their cellular source.

Before starting this study, we hypothesized that MSCs obtained from different cellular sources would exhibit different differentiation potential according to their source because cell microenvironments are one of the most important factors for differentiation. In our in vitro differentiation study, BM-MSCs were most likely to result in adipogenic, osteogenic and chondrogenic differentiation. To elucidate functional regulator that govern the differing differentiation potential of MSCs, we selected five extremely differentially expressed proteins (namely, CBR1, OAT, HSP27, GIPC1, and PSAT1) and verified their expression pattern in undifferentiated and differentiated states using western blotting and real-time RT-PCR. In the undifferentiating state, CBR 1 was extremely up-regulated in BM-MSCs, whereas OAT and GIPC were dramatically up-regulated in PB-MSCs. Additionally, HSP 27 was up-regulated in BM-MSCs and CB-MSCs, and PSAT was up-regulated in BM-MSCs and PB-MSCs. Interestingly, when MSCs differentiated into adipogenic cells, these proteins were all highly expressed in BM-MSC-derived adipocytes as compared to CB-MSC-derived adipocytes. PB-MSC-derived adipocytes showed the lowest expression of all five proteins. However, these proteins were all highly expressed in PB-MSC-derived osteocytes when inducing osteocyte differentiation as compared to CB-MSC-derived osteocytes. BM-MSC-derived osteocytes showed the lowest expression of all five proteins. In other words, the expression profiles were consistently reversed when inducing adipogenic or osteogenic differentiation, despite being irregular in the undifferentiated state. This means that the proteins involved in the differentiation mechanism function in a different manner according to their cellular source and final destination.

Although functions with respect to oxidoreductase, visual perception, stress resistance, actin-binding or biosynthesis have previously been defined, but these functions have not been described in MSCs, particularly with regard to their roles in MSC biology [26], [27], [28], [29], [30]. Recent data have demonstrated for the first time the implication of CBR1, OAT, HSP27, GIPC1 and PSAT1 in the differentiation of MSCs to both adipogenic and osteogenic lineages.

These results should provide a new direction for research focused on the development of potential therapeutic pathways using proteomics.

Furthermore, by focusing on another aspect of differentiation, we showed that the differentiation rate was highest in BM-MSCs in both adipogenic and osteogenic differentiation; the relationship between differentiation rates and expression patterns of CBR1, OAT, HSP27, GIPC1 and PSAT1 may suggest another interpretation. When BM-MSCs were differentiated into adipocytes or osteocytes, the expressions of CBR1, OAT, HSP27, GIPC1 and PSAT1 were the highest and lowest in BM-MSC-derived differentiated adipocytes and osteocytes, respectively. This could mean that CBR1, OAT, HSP27, GIPC1 and PSAT1 up-regulate adipogenic differentiation and down-regulate osteogenic differentiation in BM-MSCs. In addition, if CBR1, OAT, HSP27, GIPC1 and PSAT1 were manipulated artificially in vitro in other cell types of MSC, one might be able to regulate differentiation into adipocytes or osteocytes, much like BM-MSCs.

During immuno-staining analysis used to verify the expression localization, it was discovered that PSAT 1 is normally expressed in the cytoplasm and nucleus. In this study, the expression of PSAT was more concentrated in the nucleus of CB-MSCs as compared to BM-MSCs or PB-MSCs. In other words, cellular localization was more directed toward the cytoplasm in PB-MSCs after differentiating into osteocytes as compared to that in the undifferentiated state. These localization changes in specific proteins could also be a valuable clue for regulating differentiation into specific cell lineages.

Finally, there is a need for more additional studies investigating about the mechanisms which affect to healing process and these key regulatory factors during differentiation. The findings of the present study suggested that the different in vivo functions of MSCs may depend on their specific protein expression profile and their expressed location, and their differentiation potential could control key regulatory factors, such as CBR1, OAT, HSP27, GIPC1 and PSAT1, in different manners according to their cellular source and final destination.

In conclusion, we have demonstrated significant differences in the properties of MSCs depending on their cell source beyond donor and experimental variation. These results provide important information for selecting the optimal mesenchymal tissue as a source of cells and for enhancing clinical utility. For instance, CB-MSCs may be more appropriate for treatments aimed at increasing revascularization than PB-MSCs.

In this report, we presented for the first time large datasets of proteins involved in the mechanisms and pathways that regulate stem cell differentiation using a proteomic approach. The insights obtained in stem cell biology should provide many opportunities to improve public health. That is, this study of stem cell biology using proteomic analysis should contribute greatly to gaining insight into stem cell functioning and behavior. The results should provide clues for how to improve the cellular effect of transplantation in the field of regenerative medicine.

## Materials and Methods

### Isolation and culture of human MSCs from cord blood, peripheral blood and bone marrow

Human umbilical cord blood and peripheral blood samples were collected in NH Sodium Heparin–coated tubes (Greiner Bio-One, Kremsmunster, Austria) from healthy volunteers. BM-MSCs were purchased from Cambrex (Lonza Walkersville, Inc, MD, U.S.A.). Blood was diluted 1∶3 with D-PBS (Invitrogen, Carlsbad, CA) and overlaid onto Ficoll-Paque (Amersham, Piscataway, NJ). Cells were centrifuged at 2500 rpm for 30 minutes. Buffy coat mononuclear cells (MNCs) were collected and washed three times in D-PBS. MNCs were resuspended in MEM alpha medium (Gibco, New York, NY, U.S.A.) supplemented with glutamax (Gibco), penicillin (Gibco), streptomycin (Gibco), and 10% fetal bovine serum (Gibco). Cells were seeded on 10 cm culture dishes (Nunc, Roskilde, Denmark). After 4 days of culture, nonadherent cells and debris were aspirated, and MEM alpha medium was added. The medium was changed after 6 days. We used general cell passaging procedure using trypsinization and passage 3–5 of BM-, CB- and PB-MSCs were used in all these experiments.

In derivation, the initial seeding MNC number was 4.285×106 cells / T-75 flask in average.

### RT-PCR and quantitative real-time RT-PCR analysis

Total RNA was isolated using the Trizol reagent (Invitrogen). RNA (1 µg) was reverse-transcribed into cDNA using maxime RT premix (Intron) according to the manufacturer's instruction. In brief, the reverse transcription reaction was carried out in a 20 ul mixture at 45°C for 60 minutes. Standard PCR conditions included 2 minutes at 95°C, followed by 22–28 cycles of 30 seconds of denaturing at 95°C, then 30 seconds of annealing at 55°C and 1 minute of extension at 72°C. Primer sequences are listed in Table S1.

For real-time RT-PCR analysis, total RNA and cDNA were prepared with the same method as in the RT-PCR analysis. Real-time quantitative RT-PCR primers were targeted to adipogenic marker PPARγ2, osteogenic marker ALP (alkaline phosphatase), CRB1, GIPC1, OAT, PSAT1 and HSP27. Primer sequences are listed in Table S2.

Quantification was performed using SyBr Green gene expression assays (Roche, Mannheim, Germany). The level of target gene expression was determined by the comparative Ct method, whereby the target is normalized to the endogenous reference β-actin. The Ct value is the cycle number at which the fluorescence level reaches a given threshold. ΔCt is determined by subtracting the Ct value of the β-actin control from the Ct value of target gene [ΔCt  =  Ct (target) –Ct (β-actin)]. This relative value of target to endogenous reference is described as the fold of β-actin  = 2^−ΔCt^.

### 2-dimensional electrophoresis (2-DE) analysis and protein identification

CB-MSCs, PB-MSCs and BM-MSCs were prepared for protein extraction at passage 5 and cultured in MEM alpha medium (Gibco, New York, NY, U.S.A.) supplemented with glutamax (Gibco), penicillin (Gibco), streptomycin (Gibco), and 10% fetal bovine serum (Gibco). Pro-Prep Protein Extraction Solution (iNtRON Biotechnology) was added to each sample, and total quantity of protein (300 μg) for the analytical runs was transferred into IPG strip holder channels (Bio-Rad). 2-DE protein mixtures were separated by IEF in the first dimension and SDS-PAGE in the second dimension. Total proteins were mixed with rehydration solution (7 M urea, 2 M thiourea, 4% (w/v) CHAPS, 50 M DTT and a trace of bromophenol blue) for a final volume of 300 µl and the incubated for 12 h at RT before separation by IEF at 250 V for 15 min, 1,000 V for 2 h, or 1,000 V for 6 h, with 50 mA per gel strip. The gel strips were then immediately equilibrated in equilibrium buffer (50 mM Tris-HCl, pH 8.8, 6 M urea, 30% (v/v) glycerol, and 2% (w/v) SDS). After equilibration, the IPG gel strips were transferred for the second dimension onto SDS-PAGE followed by electrophoresis in a Protean II xi 2-DE cell (Bio-Rad) at 20 mA.

The 2-DE gels were stained using a Silver Staining Kit. Briefly, the gels were fixed in 40% ethanol and 10% acetic acid for 30 min and then sensitized in a solution of 25% (w/v) ethanol glutaraldehyde, 5% (w/v) sodium thiosulfate and 17 g of sodium acetate for 30 min. Finally, they were washed three times with water for 15 min each time. The gels were subsequently immersed in 2.5% (w/v) silver nitrate and 37% (w/v) formaldehyde for 20 min and then developed in a mixture of 6.25 g of sodium carbonate and 37% (w/v) formaldehyde for 2–5 min. The reaction was then stopped in EDTA-Na_2_-2H_2_O.

The silver-stained gels were scanned with an ImageScanner (Amersham, USA) and analyzed with Phoretix Expression software (ver. 2005; Nonlinear Dynamics, UK). Destaining and in-gel tryptic digestion of the protein spots were performed as described [31].

### Ethics

Human umbilical cord blood and peripheral blood samples were donated from healthy volunteers in CHA general hospital. The research was approved by the Korean Ministry of Health and Welfare (national approval number 86) under the Bioethics Law. On the basis of this national approval, our work was re-approved for the use of cord blood and peripheral blood by the IRB of CHA University.

### Animals and wound model

All animal procedures were approved under the guidelines of the Health Sciences Animal Policy and Welfare Committee of the CHA University College of Medicine.

Balb/C nude mice (6–8 weeks old, male, bodyweight 25–30 g) were anesthetized with an intraperitoneal injection of 100 μ*l* of a solution containing 2.22 mg ketamine and 0.17 mg xylazine. After disinfecting the dorsal surface of each mouse with an alcohol swab, two 6-mm full-thickness excisional skin wounds were created on both sides of the dorsal surface using a 6-mm biopsy punch device. Each wound received 3×10^6^ cells/ml of BM-, CB- and PB-MSCs or control vehicle medium through subcutaneous injection around the wound.

Each group underwent experiments to evaluate all wounds at seven time points for14 days post-surgery (n = 6 at each time point for each group).

### Wound analysis

Digital photographs of the wounds were taken for 14 days. The time to wound closure was defined as the time at which the wound bed was completely re-epithelialized and filled with new tissue. The wound area was measured by tracing the wound margin including the scar area and was calculated using an image analysis program (Image J). The investigators who measured the samples were blinded to the group and treatment. The percentage of wound closure was calculated as (area of original wound – area of actual wound)/area of original wound ×100. The mice were sacrificed and skin and tissue were harvested from the wound area.

### Immunohistochemical analysis

Tissue specimens were fixed in 10% freshly prepared paraformaldehyde for seven days, dehydrated with a graded ethanol series, embedded in paraffin, and cut into 6-μ*m*-thick sections. These sections were deparaffinized, hydrated and stained for CK-6, CD 3 and PECAM. Staining was performed using a standard method, and all reagents were purchased from Sigma-Aldrich. Tissue sections were analyzed using light microscopy (Nikon).

### Statistical analysis

The quantitative data are expressed as the mean ± SD. The statistical analysis was performed using the t-test. A value of *P*<0.05 was considered statistically significant.

Other material and method were available in Material and Method S1.

## Supporting Information

Figure S1 **Morphological change during cell derivation of CB-MSCs and PB-MSCs.** BM-MSCs were purchased from Cambrex to use as a representative control for MSCs. Immediately after isolation of CB-MSCs and PB-MSCs using Ficoll reagent, at day 1, only a few single cells attached to the culture dish did not form any adherent colonies. After 14 days, adherent colony forming cells appeared (passage 0), and proliferating MSCs exhibited fibroblastic and spindle-like morphology after passage 2. This was the same as purchased BM-MSCs.
(TIF)

Figure S2 **Cell growth curves of BM-, CB- and PB-MSCs.** Cell growth was recorded by ECICs (Electric Cell-substrate Impedance Sensing, Applied BioPhysics) for 40 hrs. A: BM-MSC, B:CB-MSC, C:PB-MSC, D: BM-MSC, E:CB-MSC, F:PB-MSC.
(TIF)

Material and Method S1 (DOC)

Table S1 **Primers pairs used in RT-PCR analysis.**
(DOCX)

Table S2 **Primers pairs used in Real-time RT-PCR analysis.**
(DOCX)

Table S3 **Up-regulated molecules in BM-MSCs.**
(DOCX)

Table S4 **Up-regulated molecules in CB-MSCs.**
(DOCX)

Table S5 **Up-regulated molecules in PB-MSCs.**
(DOCX)
