# Supplementary figures and images for: Proteomic Validation of Multifunctional Molecules in Mesenchymal Stem Cells Derived from Human Bone Marrow, Umbilical Cord Blood and Peripheral Blood
Source: PLoS One. 2012 May 16;7(5):e32350. doi: 10.1371/journal.pone.0032350 (PMC3353928; doi:10.1371/journal.pone.0032350)

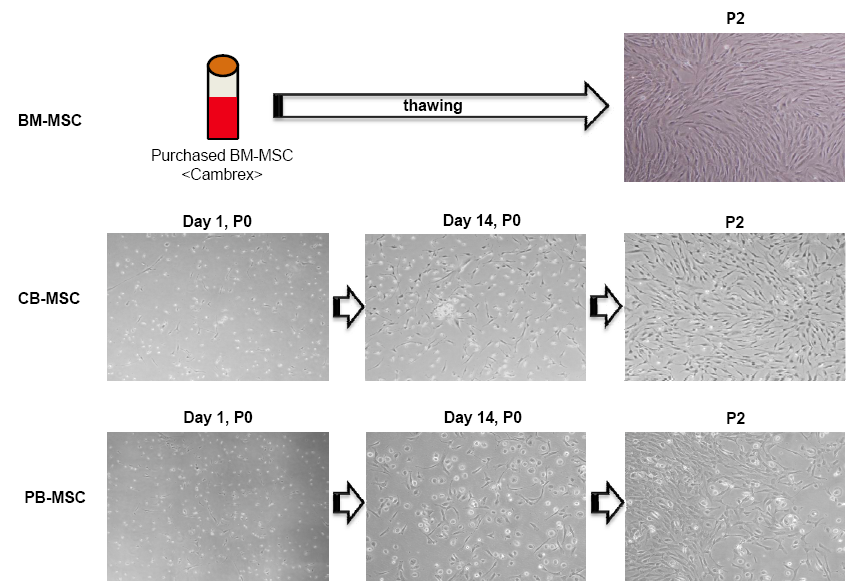

Supplement: Figure S1 — Morphological change during cell derivation of CB-MSCs and PB-MSCs. BM-MSCs were purchased from Cambrex to use as a representative control for MSCs. Immediately after isolation of CB-MSCs and PB-MSCs using Ficoll reagent, at day 1, only a few single cells attached to the culture dish did not form any adherent colonies. After 14 days, adherent colony forming cells appeared (passage 0), and proliferating MSCs exhibited fibroblastic and spindle-like morphology after passage 2. This was the same as purchased BM-MSCs. (TIF) [file pone.0032350.s001.tif]

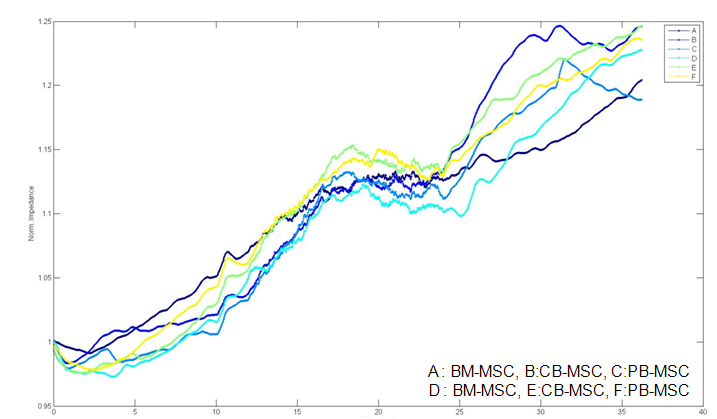

Supplement: Figure S2 — Cell growth curves of BM-, CB- and PB-MSCs. Cell growth was recorded by ECICs (Electric Cell-substrate Impedance Sensing, Applied BioPhysics) for 40 hrs. A: BM-MSC, B:CB-MSC, C:PB-MSC, D: BM-MSC, E:CB-MSC, F:PB-MSC. (TIF) [file pone.0032350.s002.tif]
