# Supplementary material for: Proteomic Validation of Multifunctional Molecules in Mesenchymal Stem Cells Derived from Human Bone Marrow, Umbilical Cord Blood and Peripheral Blood
Source: PLoS One. 2012 May 16;7(5):e32350. doi: 10.1371/journal.pone.0032350 (PMC3353928; doi:10.1371/journal.pone.0032350)
Supplement: Material and Method S1 — (DOC) [file pone.0032350.s003.doc]

**Material and Method S1**

**Cell surface-marker analysis**

The surface expression of mesenchymal stem cell markers presented on BM-, CB- and PB-MSCs was determined by flow cytometry. MSCs were dissociated with 0.25% Trypsin-EDTA (Gibco) and then washed with PBS containing 2% fetal bovine serum. They were incubated with either unstained control or antigen-specific antibodies for 20 min at RT, including CD34-PE (BD Pharmingen), CD45(R&D Systems), CD133 (BD Pharmingen), CD90 (R&D Systems) and CD44 (R&D Systems). Cells were washed twice in PBS containing 2% fetal bovine serum and resuspended in FACS buffer (2% FBS in PBS). FACS analysis was performed using a FACS Caliber FlowCytometer (BD Biosciences) and analyzed using CellQuest-Pro (BD Bioscience) software.

**Adipogenic, osteogenic and chondrogenic differentiation of MSCs from three different cellular sources**

To induce adipogenic, osteogenic and chondrogenic differentiation, CB-, PB- and BM-MSCs were grown to confluence on tissue-culture-treated dishes in an MEM alpha containing 10% FBS for one day. The next day, the medium, which was purchased from GIBCO, was changed to induce differentiation into adipocyte, osteocyte and chondrocyte. The medium was changed every 3 days. After 4 weeks of adipogenic differentiation, the differentiation potential of each of the MSCs was confirmed, and cells were fixed with 4% paraformaldehyde. They were then stained with Oil Red O and Von Kassa staining sequentially. After 4 weeks, to confirm the chondrogenic differentiation potential of each of the MSCs, after 3 weeks, 5 µm paraffin-embedded sections of pellet-cultured MSCs were stained with Alcian blue for histologic assessment after 4 weeks. Images were captured using a Nikon ECLIPSE 90i (Nikon, Japan).

## In-gel protein digestion

Protein bands of interest were excised and digested in-gel with sequencing grade, modified trypsin (Promega, Madison, WI, USA) as previously described (Bahk et al., 2004). In brief, each protein spot was excised from the gel, placed in a polypropylene (Eppendorf) tube and washed 4 to 5 times until the gel was clear with 150 µl of 1:1 acetonitrile/25 mM ammonium bicarbonate with a pH of 7.8. The gel slices were dried in a Speedvac concentrator and then rehydrated in 30 µl with 25 mM ammonium bicarbonate with a pH and 7.8 and containing 20 ng of trypsin. After incubation at 37℃ for 20 hr, the liquid was transferred to a new tube. Tryptic peptides remaining in the gel matrix were extracted for 40 min at 30℃ with 20 µl of 50% (v/v) aqueous acetonitrile containing 0.1% (v/v) formic acid. The combined supernatants were evaporated in a Speedvac concentrator and dissolved in 8 µl of 5% (v/v) aqueous acetonitrile solution containing 0.1% (v/v) formic acid for mass spectrometric analysis.

**Identification of proteins using LC-MS/MS**

The resulting tryptic peptides were separated and analyzed using reversed-phase capillary HPLC directly coupled to a Finnigan LCQ ion trap mass spectrometer (LC-MS/MS), following the procedure described by Zuo et al. (2001), with a slight modification. Both a 0.1 ⅹ 20 mm trapping and a 0.075 ⅹ 130mm resolving column were packed with Vydac 218MS low trifluoroactic acid C18 beads (5 µm in size, 300Å in pore size; Vydac, Hesperia, CA, USA) and placed in-line. Next, the peptides were bound to the trapping column for 10 min with 5% (v/v) aqueous acetonitrile containing 0.1% (v/v) formic acid, and then the bound peptides were eluted with a 50-min gradient of 5% to 80% (v/v) acetonitrile containing 0.1% (v/v) formic acid at a flow rate of 0.2 µl/min. For tandem mass spectrometry, the full mass scan range mode was set at m/z = 450 to 2000 Da. After determining the charge states of ion zoom scans, product ion spectra were acquired in MS/MS mode with a relative collision energy of 55%.

The individual spectra from MS/MS were processed using the TurboSEQUEST software (Thermo Quest, San Jose, CA). The generated peak list files were used to query either the MSDB or NCBI databases using the MASCOT program (http://www.matrixscience.com). We took into account modifications of methionine and cysteine, peptide mass tolerance at 2 Da, MS/MS ion mass tolerance at 0.8 Da, allowance of missed cleavage at 2, and charge states (namely, +1, +2 and +3). Only significant hits as defined by MASCOT probability analysis were considered initially.

**Western blotting**

To confirm the protein expression of targeted proteins, immunoblotting was performed by modifying procedures described previously ^24^. In brief, protein extracts were separated using SDS-PAGE and electro-transferred onto PVDF membranes. Samples were probed with primary antibody to detecting carbonyl reductase 1(1:1000), OAT(1:1000) , HSP27(1:1000) , GIPC1(1:1000) and PSAT1(1:1000).

After a washing with TBST, membranes were incubated with secondary antibodies for 1 h at 37°C and visualized by enhanced chemiluminescence (Amersham Biosciences). The membrane was then scanned, and the signal intensity of each band was determined using LAS 3000 (Fuji Photo Film Co., Ltd). Relative protein levels in each sample were then normalized to β-tubulin. All antibodies were purchased from Santa Cruz Biotechnology

**Immunocytochemistry**

Cells were fixed at 4% paraformaldehyde for 30 minutes and permeabilized with 0.1% Triton X-100 in PBS for 5 minutes. After blocking with 3% bovine serum albumin for 1 hour, the cells were incubated with primary antibodies at 4°C overnight. The commercial antibodies used for immunocytochemistry were goat anti-CBR1 (1:500), goat anti-OAT (1:500), goat anti-HSP27 (1:700), mouse anti-GIPC1 (1:500), and rabbit anti-PSAT1 (1:200). All antibodies were purchased from Santa Cruz Biotechnology. After being washed with PBS, the cells were incubated with secondary antibodies conjugated with Alexa 594 (Molecular Probes, U.S.A) and visualized with confocal microscopy (LSM510; Zeiss) after counterstaining with 2 µg/ml DAPI (Sigma, U.S.A).
