# Supplementary material for: Proteomic Validation of Multifunctional Molecules in Mesenchymal Stem Cells Derived from Human Bone Marrow, Umbilical Cord Blood and Peripheral Blood
Source: PLoS One. 2012 May 16;7(5):e32350. doi: 10.1371/journal.pone.0032350 (PMC3353928; doi:10.1371/journal.pone.0032350)
Supplement: Table S1 — Primers pairs used in RT-PCR analysis. (DOCX) [file pone.0032350.s004.docx]

Table S1. Primers pairs used in RT-PCR analysis

| **Gene** | **Forward primer** | **Reverse primer** | **Product size (bp)** |
| --- | --- | --- | --- |
| **CD90** | **CCCAGTGAAGATGCAGGTTT** | **GACAGCCTGAGAGGGTCTTG** | **185** |
| **CD44** | **AAAGCAGGACCTTCATCCCAGTGA** | **ATTTCCTGAGACTTGCTGGCCTCT** | **421** |
| **CD45** | **GCAAAGATGCCCAGTGTTCCACTT** | **ATCTGAGGTGTTCGCTGTGATGGT** | **474** |
| **CD133** | **CAGTCTGACCAGCGTGAAAA** | **GGCCATCCAAATCTGTCCTA** | **180** |
| **β-actin** | **TCATGACCACAGTCCATGCCATCA** | **CCCTGTTGCTGTAGCCAAATTCGT** | **450** |
