# Supplementary material for: Proteomic Validation of Multifunctional Molecules in Mesenchymal Stem Cells Derived from Human Bone Marrow, Umbilical Cord Blood and Peripheral Blood
Source: PLoS One. 2012 May 16;7(5):e32350. doi: 10.1371/journal.pone.0032350 (PMC3353928; doi:10.1371/journal.pone.0032350)
Supplement: Table S2 — Primers pairs used in Real-time RT-PCR analysis. (DOCX) [file pone.0032350.s005.docx]

Table S2. Primers pairs used in Real-time RT-PCR analysis

| **Gene** | **Forward primer** | **Reverse primer** | **Product size (bp)** |
| --- | --- | --- | --- |
| **CBR1** | **AAAGAAGGGAGTGCACCAGAAGGA** | **AGCAGGCATTCAGGAGGATCTTGT** | **143** |
| **OAT** | **GGATCCAAATGTGGCTGCGTTCAT** | **AACAGCCAGCCATCTACCAGTTCT** | **175** |
| **HSP27** | **ACGCAGTCCAACGAGATCACCAT** | **TTACTTGGCGGCAGTCTCATCGGATT** | **99** |
| **GIPC1** | **TCTGCCCTCTGCCTTTGAAGAGAA** | **TTTGTCCTTTCCCAGCTCCACCAT** | **118** |
| **PSAT1** | **TTGCTGGTGCCCAGAAGAATGTTG** | **TGTACAAGGAGCTGTTTCCAGCCT** | **138** |
| **ALP** | **ACAAGCACTCCCACTTCATCTGGA** | **TCACGTTGTTCCTGTTCAGCTCGT** | **126** |
| **PPARγ2** | **AGCCTCATGAAGAGCCTTCCAACT** | **TCCGGAAGAAACCCTTGCATCCTT** | **120** |
| **β-actin** | **TGGATCAGCAAGCAGGAGTATG** | **AAGAAAGGGTGTAACGCAACTAAG** | **98** |
