# Supplementary material for: Proteomic Validation of Multifunctional Molecules in Mesenchymal Stem Cells Derived from Human Bone Marrow, Umbilical Cord Blood and Peripheral Blood
Source: PLoS One. 2012 May 16;7(5):e32350. doi: 10.1371/journal.pone.0032350 (PMC3353928; doi:10.1371/journal.pone.0032350)
Supplement: Table S3 — Up-regulated molecules in BM-MSCs. (DOCX) [file pone.0032350.s006.docx]

Table S3. Up-regulated BM-MSCs

| Spot | Accession | Identified proteins | Seq. | Matched | pI | Mass | Mascot |
| --- | --- | --- | --- | --- | --- | --- | --- |
| No. | No. |  | Cov. (%) | peptide |  | (Da) | Score |
| 4 | IPI00218343 | Tubulin alpha-1C chain | 8 | 3 | 4.96 | 50560 | 119 |
| 10 | [IPI00930609](http://210.219.44.156/mascot/cgi/protein_view.pl?file=../data/20091127/F002729.dat&hit=IPI00930609&px=1&_server_mudpit_switch=0.001) | Isoform 1 of Phosphoserine aminotransferase | 37 | 20 | 7.56 | 40803 | 400 |
| 11 | [IPI00930609](http://210.219.44.156/mascot/cgi/protein_view.pl?file=../data/20091127/F002731.dat&hit=IPI00930609&px=1&_server_mudpit_switch=0.001) | Isoform 1 of Phosphoserine aminotransferase | 15 | 5 | 7.56 | 40803 | 137 |
| 12 | [IPI00930609](http://210.219.44.156/mascot/cgi/protein_view.pl?file=../data/20091127/F002733.dat&hit=IPI00930609&px=1&_server_mudpit_switch=0.001) | Isoform 1 of Phosphoserine aminotransferase | 37 | 21 | 7.56 | 40803 | 349 |
| 16 | [IPI00295386](http://210.219.44.156/mascot/cgi/protein_view.pl?file=../data/20091130/F002768.dat&hit=IPI00295386&px=1&_server_mudpit_switch=0.001) | Carbonyl reductase [NADPH] 1 | 51 | 15 | 8.55 | 30646 | 402 |
| 18 | [IPI00455315](http://210.219.44.156/mascot/cgi/protein_view.pl?file=../data/20091130/F002773.dat&hit=IPI00455315&px=1&_server_mudpit_switch=0.001) | Annexin A2 | 62 | 31 | 7.57 | 38812 | 890 |
| 20 | IPI00411706 | S-formylglutathione hydrolase | 42 | 18 | 6.54 | 31965 | 399 |
| 25 | [IPI00020906](http://210.219.44.156/mascot/cgi/protein_view.pl?file=../data/20091130/F002786.dat&hit=IPI00020906&px=1&_server_mudpit_switch=0.001) | Inositol monophosphatase | 29 | 6 | 5.16 | 30575 | 152 |
| 26 | [IPI00384051](http://210.219.44.156/mascot/cgi/protein_view.pl?file=../data/20091130/F002788.dat&hit=IPI00384051&px=1&_server_mudpit_switch=0.001) | Putative uncharacterized protein PSME2 | 51 | 15 | 6.05 | 28758 | 432 |
| 28 | [IPI00027681](http://210.219.44.156/mascot/cgi/protein_view.pl?file=../data/20091130/F002792.dat&hit=IPI00027681&px=1&_server_mudpit_switch=0.001) | Nicotinamide N-methyltransferase | 24 | 7 | 5.56 | 30019 | 225 |
| 29 | [IPI00025512](http://210.219.44.156/mascot/cgi/protein_view.pl?file=../data/20091130/F002794.dat&hit=IPI00025512&px=1&_server_mudpit_switch=0.001) | Heat shock protein beta-1 | 85 | 27 | 5.98 | 22826 | 632 |
| 30 | [IPI00246975](http://210.219.44.156/mascot/cgi/protein_view.pl?file=../data/20091130/F002796.dat&hit=IPI00246975&px=1&_server_mudpit_switch=0.001) | Glutathione S-transferase Mu 3 | 47 | 15 | 5.37 | 27006 | 414 |
| 31 | [IPI00019755](http://210.219.44.156/mascot/cgi/protein_view.pl?file=../data/20091130/F002798.dat&hit=IPI00019755&px=1&_server_mudpit_switch=0.001) | Glutathione S-transferase omega-1 | 48 | 32 | 6.23 | 27838 | 577 |
