# Supplementary material for: Proteomic Validation of Multifunctional Molecules in Mesenchymal Stem Cells Derived from Human Bone Marrow, Umbilical Cord Blood and Peripheral Blood
Source: PLoS One. 2012 May 16;7(5):e32350. doi: 10.1371/journal.pone.0032350 (PMC3353928; doi:10.1371/journal.pone.0032350)
Supplement: Table S4 — Up-regulated molecules in CB-MSCs. (DOCX) [file pone.0032350.s007.docx]

Table S4. Up-regulated CB-MSCs

| Spot | Accession | Identified proteins | Seq. | Matched | pI | Mass | Mascot |
| --- | --- | --- | --- | --- | --- | --- | --- |
| No. | No. |  | Cov. (%) | peptide |  | (Da) | Score |
| 3 | [IPI00745872](http://210.219.44.156/mascot/cgi/protein_view.pl?file=../data/20091127/F002716.dat&hit=IPI00745872&px=1&_server_mudpit_switch=0.001) | Isoform 1 of Serum albumin | 7 | 10 | 5.92 | 71352 | 273 |
| 5 | [IPI00418471](http://210.219.44.156/mascot/cgi/protein_view.pl?file=../data/20091127/F002721.dat&hit=IPI00418471&px=1&_server_mudpit_switch=0.001) | Vimentin | 73 | 110 | 5.06 | 53677 | 2371 |
| 6 | [IPI00015842](http://210.219.44.156/mascot/cgi/protein_view.pl?file=../data/20091127/F002722.dat&hit=IPI00015842&px=1&_server_mudpit_switch=0.001) | Reticulocalbin-1 | 39 | 15 | 4.86 | 38866 | 420 |
| 7 | [IPI00549248](http://210.219.44.156/mascot/cgi/protein_view.pl?file=../data/20091127/F002724.dat&hit=IPI00549248&px=1&_server_mudpit_switch=0.001) | Isoform 1 of Nucleophosmin | 34 | 29 | 4.64 | 32729 | 837 |
| 15 | [IPI00011253](http://210.219.44.156/mascot/cgi/protein_view.pl?file=../data/20091130/F002766.dat&hit=IPI00011253&px=1&_server_mudpit_switch=0.001) | 40S ribosomal protein S3 | 73 | 37 | 9.68 | 26845 | 845 |
| 17 | [IPI00218918](http://210.219.44.156/mascot/cgi/protein_view.pl?file=../data/20091130/F002771.dat&hit=IPI00218918&px=1&_server_mudpit_switch=0.001) | Annexin A1 | 54 | 33 | 6.57 | 38922 | 1154 |
| 21 | [IPI00411706](http://210.219.44.156/mascot/cgi/protein_view.pl?file=../data/20091130/F002778.dat&hit=IPI00411706&px=1&_server_mudpit_switch=0.001) | S-formylglutathione hydrolase | 38 | 17 | 6.54 | 31965 | 410 |
| 27 | [IPI00001960](http://210.219.44.156/mascot/cgi/protein_view.pl?file=../data/20091130/F002790.dat&hit=IPI00001960&px=1&_server_mudpit_switch=0.001) | Chloride intracellular channel protein 4 | 33 | 11 | 5.45 | 28986 | 144 |
| 29 | [IPI00025512](http://210.219.44.156/mascot/cgi/protein_view.pl?file=../data/20091130/F002794.dat&hit=IPI00025512&px=1&_server_mudpit_switch=0.001) | Heat shock protein beta-1 | 85 | 27 | 5.98 | 22826 | 632 |
| 30 | [IPI00246975](http://210.219.44.156/mascot/cgi/protein_view.pl?file=../data/20091130/F002796.dat&hit=IPI00246975&px=1&_server_mudpit_switch=0.001) | Glutathione S-transferase Mu 3 | 47 | 15 | 5.37 | 27006 | 414 |
| 31 | [IPI00019755](http://210.219.44.156/mascot/cgi/protein_view.pl?file=../data/20091130/F002798.dat&hit=IPI00019755&px=1&_server_mudpit_switch=0.001) | Glutathione S-transferase omega-1 | 48 | 32 | 6.23 | 27838 | 577 |
