# Supplementary material for: Proteomic Validation of Multifunctional Molecules in Mesenchymal Stem Cells Derived from Human Bone Marrow, Umbilical Cord Blood and Peripheral Blood
Source: PLoS One. 2012 May 16;7(5):e32350. doi: 10.1371/journal.pone.0032350 (PMC3353928; doi:10.1371/journal.pone.0032350)
Supplement: Table S5 — Up-regulated molecules in PB-MSCs. (DOCX) [file pone.0032350.s008.docx]

Table S5. Up-regulated PB-MSCs

| Spot | Accession | Identified proteins | Seq. | Matched | pI | Mass | Mascot |
| --- | --- | --- | --- | --- | --- | --- | --- |
| No. | No. |  | Cov. (%) | peptide |  | (Da) | Score |
| 1 | [IPI00217975](http://210.219.44.156/mascot/cgi/protein_view.pl?file=../data/20091127/F002713.dat&hit=IPI00217975&px=1&_server_mudpit_switch=0.001) | Lamin-B1 | 49 | 42 | 5.11 | 66658 | 1008 |
| 2 | [IPI00167949](http://210.219.44.156/mascot/cgi/protein_view.pl?file=../data/20091127/F002714.dat&hit=IPI00167949&px=1&_server_mudpit_switch=0.001) | Interferon-induced GTP-binding protein Mx1 | 42 | 35 | 5.6 | 75893 | 665 |
| 4 | IPI00218343 | Tubulin alpha-1C chain | 8 | 3 | 4.96 | 50560 | 119 |
| 5 | [IPI00418471](http://210.219.44.156/mascot/cgi/protein_view.pl?file=../data/20091127/F002721.dat&hit=IPI00418471&px=1&_server_mudpit_switch=0.001) | Vimentin | 73 | 110 | 5.06 | 53677 | 2371 |
| 6 | [IPI00015842](http://210.219.44.156/mascot/cgi/protein_view.pl?file=../data/20091127/F002722.dat&hit=IPI00015842&px=1&_server_mudpit_switch=0.001) | Reticulocalbin-1 | 39 | 15 | 4.86 | 38866 | 420 |
| 7 | [IPI00549248](http://210.219.44.156/mascot/cgi/protein_view.pl?file=../data/20091127/F002724.dat&hit=IPI00549248&px=1&_server_mudpit_switch=0.001) | Isoform 1 of Nucleophosmin | 34 | 29 | 4.64 | 32729 | 837 |
| 8 | [IPI00022334](http://210.219.44.156/mascot/cgi/protein_view.pl?file=../data/20091127/F002726.dat&hit=IPI00022334&px=1&_server_mudpit_switch=0.001) | Ornithine aminotransferase, mitochondrial | 32 | 12 | 6.57 | 48852 | 151 |
| 9 | [IPI00024705](http://210.219.44.156/mascot/cgi/protein_view.pl?file=../data/20091127/F002727.dat&hit=IPI00024705&px=1&_server_mudpit_switch=0.001) | PDZ domain-containing protein GIPC1 | 13 | 3 | 5.9 | 36143 | 155 |
| 10 | [IPI00930609](http://210.219.44.156/mascot/cgi/protein_view.pl?file=../data/20091127/F002729.dat&hit=IPI00930609&px=1&_server_mudpit_switch=0.001) | Isoform 1 of Phosphoserine aminotransferase | 37 | 20 | 7.56 | 40803 | 400 |
| 11 | [IPI00930609](http://210.219.44.156/mascot/cgi/protein_view.pl?file=../data/20091127/F002731.dat&hit=IPI00930609&px=1&_server_mudpit_switch=0.001) | Isoform 1 of Phosphoserine aminotransferase | 15 | 5 | 7.56 | 40803 | 137 |
| 12 | [IPI00930609](http://210.219.44.156/mascot/cgi/protein_view.pl?file=../data/20091127/F002733.dat&hit=IPI00930609&px=1&_server_mudpit_switch=0.001) | Isoform 1 of Phosphoserine aminotransferase | 37 | 21 | 7.56 | 40803 | 349 |
| 14 | [IPI00215965](http://210.219.44.156/mascot/cgi/protein_view.pl?file=../data/20091130/F002764.dat&hit=IPI00215965&px=1&_server_mudpit_switch=0.001) | Isoform A1-B of Heterogeneous nuclear ribonucleoprotein A1 | 4 | 3 | 9.26 | 38938 | 35 |
| 15 | [IPI00011253](http://210.219.44.156/mascot/cgi/protein_view.pl?file=../data/20091130/F002766.dat&hit=IPI00011253&px=1&_server_mudpit_switch=0.001) | 40S ribosomal protein S3 | 73 | 37 | 9.68 | 26845 | 845 |
| 19 | [IPI00455315](http://210.219.44.156/mascot/cgi/protein_view.pl?file=../data/20091130/F002774.dat&hit=IPI00455315&px=1&_server_mudpit_switch=0.001) | Annexin A2 | 39 | 14 | 8.53 | 38812 | 439 |
| 21 | [IPI00411706](http://210.219.44.156/mascot/cgi/protein_view.pl?file=../data/20091130/F002778.dat&hit=IPI00411706&px=1&_server_mudpit_switch=0.001) | S-formylglutathione hydrolase | 38 | 17 | 6.54 | 31965 | 410 |
| 22 | [IPI00217966](http://210.219.44.156/mascot/cgi/protein_view.pl?file=../data/20091130/F002780.dat&hit=IPI00217966&px=1&_server_mudpit_switch=0.001) | L-lactate dehydrogenase | 17 | 6 | 8.61 | 40102 | 132 |
| 23 | [IPI00303602](http://210.219.44.156/mascot/cgi/protein_view.pl?file=../data/20091130/F002782.dat&hit=IPI00303602&px=1&_server_mudpit_switch=0.001) | Isoform 1 of Axin interactor, dorsalization-associated protein | 9 | 3 | 6.13 | 35175 | 82 |
| 24 | [IPI00021439](http://210.219.44.156/mascot/cgi/protein_view.pl?file=../data/20091130/F002784.dat&hit=IPI00021439&px=1&_server_mudpit_switch=0.001) | Actin, cytoplasmic 1 | 11 | 4 | 5.29 | 42058 | 61 |
